# O.2.2-2 An intervention study design to increase physical activity and health using outdoor spaces: the GoGreenRoutes project

**DOI:** 10.1093/eurpub/ckad133.117

**Published:** 2023-09-11

**Authors:** Thayse Natacha Gomes, Sara Suikkanen, Triin Rääsk, Saima Kuu, Ilkka Väänänen, Alan Donnelly

**Affiliations:** Department of Physical Education and Sport Sciences, Physical Activity for Health Research Cluster, Health Research Institute, University of Limerick; Physical Activity and Functional Capacity Research Group, Faculty of Health Care, LAB University of Applied Sciences; Institute of Natural and Health Sciences, Tallinna University; School of Natural Sciences and Health, Tallinn University; thayse.gomes@ul.ie; Physical Activity and Functional Capacity Research Group, Faculty of Health Care, LAB University of Applied Sciences; Department of Physical Education and Sport Sciences, Physical Activity for Health Research Cluster, Health Research Institute, University of Limerick

## Abstract

**Purpose:**

For outdoor physical activity (PA), it is likely that the quality of the environment is a factor which determines adherence and compliance with PA at the population level. There is some evidence that a higher environmental quality contributes to the improvement of well-being and mental health, and increased in PA. The GoGreenRoutes intervention study will explore the relationship between outdoor environmental quality and PA adherence, and determine whether environmental exposure during PA indirectly influences health.

**Project description:**

The project will be carried out in up to six cities, including Lahti (Finland), Limerick (Ireland), and Tallinn (Estonia) as part of the European Commission-funded project GoGreenRoutes. The participant population will comprise inactive adults of both sexes (aged 30-65y). The intervention will consist of an 8-week self-guided programme, where participants will perform PA in self-selected outdoor spaces (at least 3 times/week, 30min/session). This will be a two-group design, where the experimental group will be given advice and instructions in selecting a green route; while the control group, on the opposite, will be given advice and instructions in selecting an urban/road route. Both groups will receive information about the benefits of PA performed in outdoor spaces (control group) and green/nature spaces (experimental group). Environmental quality in each selected route will be assessed. Time spent in different movement behaviours will be measured at the baseline and week eight, using an activPAL accelerometer over a nine-day measurement period. Further, differences in anthropometric and physical fitness measurements, health perception and quality of life, sleep quality, and beliefs about green exercise will be investigated, as well as their associations with movement behaviours and environmental quality.

**Conclusion:**

The intervention design has been guided by results from the pilot/feasibility study. It will contribute to the understanding of the relationship between environmental quality and PA adherence, and will provide data on the effects of self-selected outdoor route environmental quality on the daily PA undertaken in the sample population.

**Funding Source:**

European Union’s Horizon 2020 research and innovation program, through the GoGreenRoutes research project under grant agreement No. 869764.

